# Old disease, new twist

**DOI:** 10.1002/jhm.70442

**Published:** 2026-09-28

**Authors:** Hadi Roudsari, Gurpreet Dhaliwal, Anand D. Jagannath, Alan H. Baik, Sam Brondfield

**Affiliations:** ^1^ Department of Medicine University of California, San Francisco San Francisco California USA; ^2^ Division of Hospital Medicine University of California, San Francisco San Francisco California USA; ^3^ San Francisco Veterans Affairs Medical Center San Francisco California USA; ^4^ Division of Hospital Medicine Oregon Health and Science University Portland Oregon USA; ^5^ Division of Cardiology University of California, San Francisco San Francisco California USA; ^6^ Division of Hematology/Oncology University of California, San Francisco San Francisco California USA; ^7^ UCSF Helen Diller Family Comprehensive Cancer Center San Francisco California USA

## Abstract

An 81‐year‐old man with a history of high‐grade urothelial carcinoma of the bladder and ureter on treatment with atezolizumab and tiragolumab presented to the emergency department with 5 months of malaise, generalized weakness, and fatigue and 2 weeks of subjective fevers. One month prior to presentation, he received an empiric 9‐day course of levofloxacin for possible epididymitis based on scrotal pain and an ultrasound showing mild bilateral epididymal prominence. Blood and urine cultures were negative. His constitutional symptoms persisted, and he developed a subjective fever 2 weeks prior to presentation. He was admitted for further evaluation.


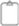
 **An 81‐year‐old man with a history of high‐grade urothelial carcinoma of the bladder and ureter on treatment with atezolizumab and tiragolumab presented to the emergency department with 5 months of malaise, generalized weakness, and fatigue and 2 weeks of subjective fevers. One month prior to presentation, he received an empiric 9‐day course of levofloxacin for possible epididymitis based on scrotal pain and an ultrasound showing mild bilateral epididymal prominence. Blood and urine cultures were negative. His constitutional symptoms persisted, and he developed a subjective fever 2 weeks prior to presentation. He was admitted for further evaluation.**


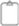
 **Atezolizumab and tiragolumab had been initiated 7 months prior to admission. Two months after starting this therapy, the bladder (stage III) and ureter (stage I) carcinomas were treated with radical cystoprostatectomy, right nephroureterectomy, and ileal conduit creation. Atezolizumab and tiragolumab were continued post‐operatively as adjuvant therapy**.


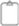
 **His medical history included stage IA left lung adenocarcinoma treated with lobectomy 9 years earlier, stage 4 chronic kidney disease (CKD) attributed to obstructive nephropathy from prostatic hypertrophy, gout, asthma, and obstructive sleep apnea.**

Chronic malaise punctuated by 2 weeks of fevers points to an infection, autoimmune condition, or malignancy. His age, kidney disease, and history of cancer increase his susceptibility to infection. The lack of improvement after 9 days of antibiotics modestly decreases the probability of an indolent bacterial infection, such as endocarditis or intra‐abdominal abscess, where a short course of antibiotics is more likely to be suppressive than curative. Mycobacterial, fungal, and parasitic infections are important considerations when fever extends for weeks. Cancer recurrence could be heralded by a fever, although this is rare.

Immune checkpoint inhibitor (ICI) therapy, which enhances the immune system's ability to recognize and attack tumor cells, can also trigger immune‐related adverse events (irAEs), which resemble autoimmune diseases. Certain irAE endocrinopathies, such as hypothyroidism or adrenal insufficiency, could account for his malaise but not his fever.


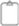
 **His home medications included an albuterol inhaler, a budesonide‐formoterol inhaler, allopurinol, vitamin B‐complex and vitamin D supplements, pravastatin, ezetimibe, coenzyme Q10, lactobacillus, and oxycodone. He quit smoking at age 30. He previously consumed wine occasionally but stopped 2 years ago. He was a retired biotechnology executive with no significant occupational or environmental exposures.**


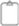
 **In the emergency department, he did not have chest pain, dyspnea, or palpitations. The vital signs were normal. Cardiopulmonary, abdominal, and neurologic examinations were normal, including examination of the ileal conduit. Jugular venous pulse was not assessed. Peripheral edema was absent. Laboratory results (and baseline from 4 weeks prior, when available) showed a white blood cell (WBC) count of 14,300/µl (9900/µl) with polymorphonuclear cell predominance, hemoglobin 12.3 g/dl (11.4 g/dl), sodium 131 mmol/L, creatinine 2.4 mg/dl (2.4 mg/dl), calcium 9.8 mg/dl, albumin 2.8 g/dl, troponin I 0.45 µg/L (normal < 0.05 µg/L), and urinalysis with trace hemoglobin, 30 mg/dl protein, and no cells. Chest radiograph showed mild bibasilar hazy opacities. Serial electrocardiograms (ECG) showed a normal sinus rhythm with no ischemic changes. A repeat scrotal ultrasound was unremarkable.**


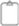
 **On day 2, the temperature increased to 39.2°C, and vancomycin and piperacillin‐tazobactam were administered. Computed tomography (CT) of the chest, abdomen, and pelvis with intravenous (IV) contrast revealed centrilobular nodules in the right upper lobe, trace bilateral pleural effusions, mild pulmonary edema, and subtle hypoenhancement of the entire left ventricle. No remarkable findings were noted in the abdomen or pelvis. The troponin increased to 0.78 µg/L and remained between 0.70 and 0.80 µg/L over the next 24 h.**

Centrilobular nodules of the lung could reflect infection, hypersensitivity, aspiration, or drug‐induced pneumonitis from an irAE. Bacterial, mycobacterial, and fungal pathogens all warrant consideration. Metastatic recurrence from the urothelial cancer is unlikely with no evidence of intraabdominal disease or lymphadenopathy.

Fever and non‐dynamic troponin elevation are seen with myocardial injury in sepsis, myocarditis (which may be infectious or immune in nature), or endocarditis. The hypoenhancement of the left ventricle may represent reduced myocardial perfusion, which can result from an ischemic macrovascular event such as acute coronary syndrome or microvascular dysfunction; however, the absence of chest pain, ischemic ECG changes, or a dynamic rise in troponin excludes acute coronary syndrome. Myocardial drug toxicity merits consideration. The CT findings of mild pulmonary edema and bilateral pleural effusions could signify new heart failure or could reflect an undefined pneumonitis.

The mild hypercalcemia (10.8 mg/dl when corrected for low albumin) should be evaluated with the parathyroid hormone (PTH) level. If PTH is suppressed, then testing should focus on cancers (e.g., multiple myeloma and parathyroid hormone‐related peptide testing), excess vitamin D, excess calcium intake, and granulomatous disorders (e.g., sarcoidosis). Interpretation of these parameters may be confounded by acute kidney injury (AKI) and CKD; the latter causes secondary hyperparathyroidism characterized by elevated PTH, low or normal calcium, and reduced 1,25‐dihydroxyvitamin D levels.


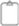
 **After 48 h, the antibiotics were changed to ceftriaxone and doxycycline to treat possible pneumonia. By hospital day 5, the fevers had abated, but malaise and generalized weakness persisted. Blood and urine cultures and respiratory viral studies were negative.**


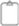
 **Laboratory results on hospital day 5 showed a WBC count of 11,900/µl, creatinine 4.4 mg/dl, calcium 10.1 mg/dl, and albumin 2.2 g/dl. Thyroid‐stimulating hormone was 1.21 mIU/L (normal 0.45–4.12 mIU/L). Morning cortisol was 19 µg/dl (normal 4‐19 µg/dl). Urinalysis, repeated due to AKI, revealed 5–10 white blood cells and 30 mg/dl protein.**


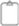
 **The B‐type natriuretic peptide (BNP) was 1900 pg/ml (normal < 79 pg/ml). An echocardiogram showed a left ventricular (LV) ejection fraction of 70%, impaired LV relaxation, moderate concentric LV hypertrophy, increased echogenicity in the inferior wall, and a trace pericardial effusion. IV furosemide was administered, leading to 1 L of urine output without clinical improvement. By hospital day 6, the creatinine had decreased to 3.9 mg/dl.**


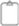
 **Given the absence of evidence of infection, resolution of fevers, and patient preference, he was discharged on hospital day 6. Prescriptions for guideline‐directed medical therapy for heart failure and diuretics were deferred, given his euvolemic status, preserved ejection fraction, and closely scheduled outpatient cardiology evaluation.**

Although hypervolemia was not evident on physical exam, the elevated troponin, BNP, and diuretic response to furosemide are consistent with decompensated heart failure with preserved ejection fraction. The presence of diastolic dysfunction and concentric LV hypertrophy raises concern for an underlying infiltrative cardiomyopathy. Increased echogenicity is a non‐specific finding but can reflect infiltrative cardiomyopathy, myocardial fibrosis, ischemic injury, or myocarditis. The AKI may have been caused by cardiorenal physiology or interstitial nephritis (suggested by the mild pyuria and proteinuria).

The approach to myocarditis entails evaluation for infections and immune conditions. Serologic and polymerase chain reaction (PCR) testing should be pursued for cardiotropic pathogens (e.g., coxsackievirus, parvovirus B19, *Borrelia burgdorferi, Trypanosoma cruzi, Toxoplasma gondii*). Leading autoimmune causes for myocarditis include sarcoidosis, systemic lupus erythematosus, and irAE. The albumin‐corrected calcium value has gone from 10.8 to 11.5 mg/dl. Hypercalcemia, cardiomyopathy, and pneumonitis are all features of sarcoidosis, although the characteristic mediastinal lymphadenopathy is absent.


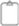
 **Two weeks after discharge, the patient was seen at the cardiology clinic, where he was noted to have weakness, anorexia, and pain and swelling in both shoulders, hands, and knees. The temperature was 37.1°C, heart rate 83 beats/min, blood pressure 117/67 mmHg, respiratory rate 15 breaths/min, and oxygen saturation 99% on room air. The cardiopulmonary and volume status exams were normal. The WBC count was 19,300/µl, creatinine 2.6 mg/dl, calcium 13 mg/dl, albumin 2.5 g/dl, ionized calcium 1.56 mmol/L (normal 1.14–1.34 mmol/L), BNP 768 pg/ml, and troponin 1 µg/L. He was readmitted to the hospital. IV fluids, calcitonin, and denosumab were administered.**

Symmetric pain and swelling in the small and large joints of the upper and lower extremities reflect polyarthritis, which is caused by a degenerative, crystalline, infectious, or autoimmune process. A viral infection like parvovirus B19 could present as polyarthritis mimicking rheumatoid arthritis. The marked leukocytosis prompts consideration of septic arthritis. Crystalline diseases are rarely perfectly symmetric. Autoimmunity is most likely to account for symmetric, progressive polyarthritis. Initial considerations would be rheumatoid arthritis, systemic lupus erythematosus, psoriatic arthritis, sarcoidosis, and irAE.

The serum creatinine has declined, but the mechanism of improvement is uncertain. If there was an element of cardiorenal syndrome during the previous hospital admission, it is conceivable that hypercalcemia (through its downregulation of aquaporin‐2 water channels in the collecting duct) coincidentally did the work of a diuretic to achieve euvolemia.


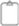
 **Blood and urine cultures were sterile, and respiratory viral studies were negative. By day 3, the WBC count was 13,500/µl, the creatinine was 2.1 mg/dl, and the calcium was 10.5 mg/dl. A repeat echocardiogram showed unchanged concentric LV hypertrophy. On day 6, he developed atrial flutter with variable block.**


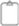
 **PTH was 7 ng/dl (normal 18–90 ng/dl), and 25‐hydroxyvitamin D was 49 ng/ml (normal 20–50 ng/ml). PTH‐related protein (PTHrP) was 0.8 pmol/L (normal < 4.3 pmol/L). 1,25‐dihydroxyvitamin D was > 150 pg/ml (normal 20–79 pg/ml). Whole‐body fluorodeoxyglucose positron emission tomography with computed tomography (FDG‐PET/CT) revealed right middle lobe ground glass opacities with radiotracer uptake as well as uptake in the synovia of multiple joints and throughout the myocardium.**

The elevated 1,25‐dihydroxyvitamin D suggests that there is excessive 1‐alpha hydroxylation of vitamin D in the macrophages of granulomas, which proliferate in autoimmune, malignant, and infectious conditions. Rarely, malignant lymphocytes can generate excess calcitriol, but the absence of lymphadenopathy or organomegaly on serial imaging makes lymphoma unlikely. Indolent infections such as mycobacteria and fungi, particularly in their disseminated forms, can generate widespread granulomas. Sarcoidosis is the prototypical autoimmune granulomatous disorder, and involvement of the lungs, myocardium, and joints in conjunction with elevated 1,25‐dihydroxyvitamin D makes it the most likely diagnosis.

The radiotracer avidity confirms an inflammatory process in the heart, such as myocarditis or sarcoidosis, further supported by the onset of a new arrhythmia. Cardiac magnetic resonance imaging (MRI) can characterize the pattern of myocardial involvement, which may help differentiate myocarditis from cardiac sarcoidosis and guide decision‐making around endomyocardial biopsy.


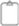
 **On day 9, after multidisciplinary discussions involving hospital medicine, cardiology, endocrinology, rheumatology, and oncology, the patient was prescribed prednisone 50 mg daily for an incompletely characterized multisystem inflammatory condition. The next day, the patient reported significant improvement in his joint swelling, pain, and weakness. On day 13, cardiac MRI revealed asymmetric thickening (up to 18 mm) of the mid‐to‐basal septum and myocardial edema. The patient's condition continued to improve, and he was able to participate in physical therapy.**

Rapid steroid responsiveness suggests interruption of an immune‐mediated process, although infections, cancers, and drug toxicities are often temporarily ameliorated by steroids as well. The pattern of organ involvement and 1,25‐dihydroxyvitamin D‐mediated hypercalcemia suggests a disseminated granulomatous disease such as sarcoidosis, which could arise idiopathically or on account of the ICI therapy. One important counterargument to the sarcoidosis hypothesis is the absence of hilar lymphadenopathy. Infection should not be discounted in this patient with fevers and leukocytosis. Myocardial or synovial biopsy and cultures would be informative.


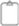
 **An FDG‐PET/CT scan with resting myocardial perfusion protocol showed abnormal uptake in the base of the lateral wall, apex, and mid‐septum, suggestive of active cardiac sarcoidosis** (Figure 1). **Endomyocardial biopsy of the right ventricle, performed to confirm the diagnosis, revealed lymphocytic infiltrates with multinucleated giant cells, most consistent with active cardiac sarcoidosis. Transthyretin amyloidosis was incidentally detected in the biopsy specimen and later confirmed by mass spectrometry** (Figure 2). **A cardiac pyrophosphate scan showed high‐grade myocardial uptake indicative of transthyretin amyloidosis. Serum free light chains, serum and urine protein electrophoresis, and serum and urine protein immunofixation were normal. Microbiologic studies were not done on the endomyocardial tissue, as clinical suspicion for cardiac sarcoidosis was high. The patient was discharged on prednisone for sarcoidosis and tafamidis for transthyretin amyloidosis.**

**Figure 1:**
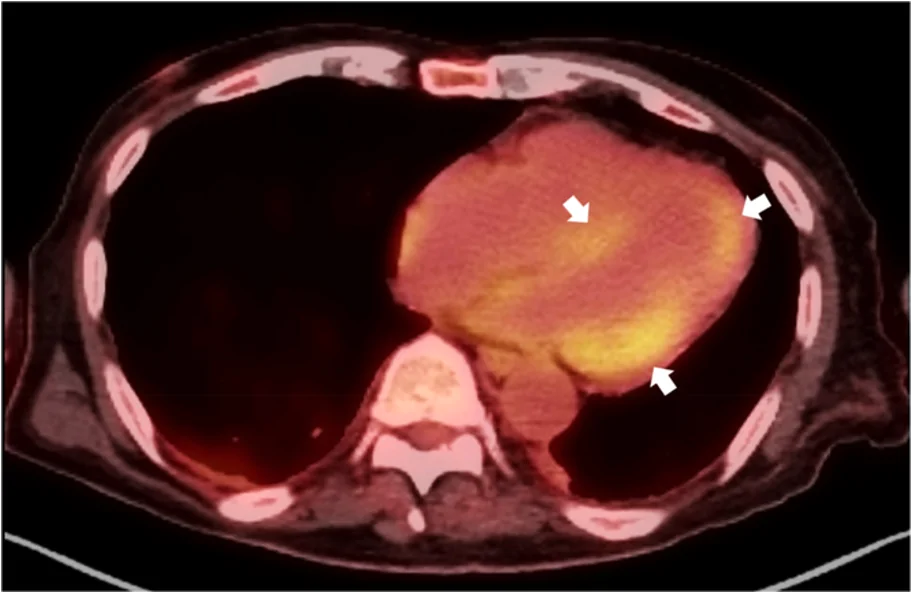
Cardiac positron emission tomography/computed tomography with 18F‐fluorodeoxyglucose demonstrates increased FDG uptake in the lateral, apical, and mid‐septal walls of the left ventricle (white arrows).

**Figure 2:**
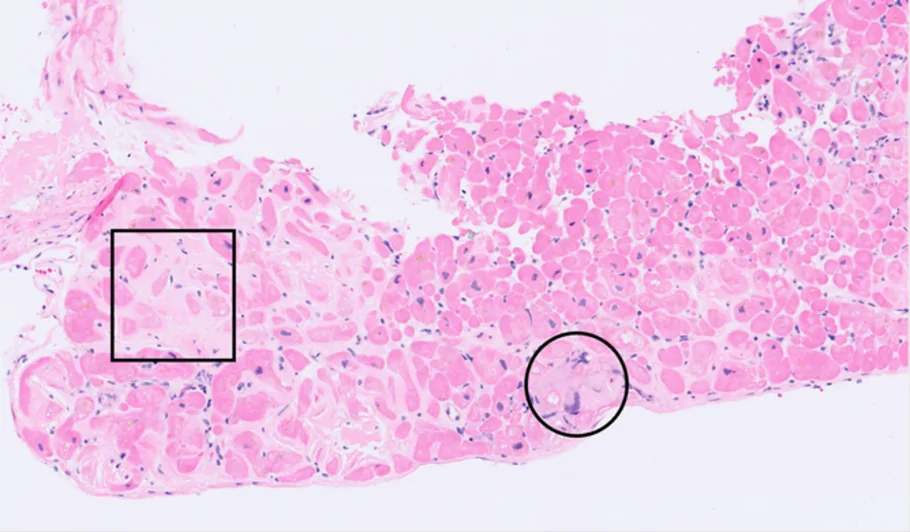
Endomyocardial biopsy demonstrating granulomatous inflammation and amyloid deposition. Multiple small foci of mononuclear interstitial inflammation are present between myocytes. A multinucleated giant cell is visible within an inflammatory focus (circle), characterized by multiple closely apposed nuclei within a single enlarged cell. Also present is amorphous eosinophilic interstitial material consistent with amyloid deposition (square). Hematoxylin and eosin stain.


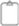
 **Serial FDG‐PET/CT imaging showed persistent disease, prompting a switch from prednisone to azathioprine and then adalimumab, with subsequent resolution of active cardiac sarcoidosis on imaging. ICI therapy was discontinued due to cardiac sarcoidosis, with close observation recommended. Subsequent imaging showed no signs of cancer recurrence. The imaging findings, endomyocardial biopsy, multi‐organ involvement, initial clinical response to corticosteroids, and temporal association with ICI therapy established the diagnosis of ICI–associated sarcoidosis with cardiac involvement.**

## DISCUSSION

Immune checkpoint inhibitors (ICI) have transformed the landscape of cancer treatment.^1^ These monoclonal antibodies enhance the immune response against tumors by blocking an inhibitory receptor or ligand on the surface of T‐lymphocytes.^2^, ^3^ However, these immunotherapies can also trigger immune‐related adverse events (irAEs) that can affect multiple organs, including the skin, endocrine organs, gastrointestinal tract, lungs, muscle, liver, and heart.^4^ These toxicities can occur at any point during ICI treatment, though they are most common within the first 3 months of therapy.^5^

The incidence and site of irAEs vary based on ICI and patient characteristics. Skin and gastrointestinal irAEs are the most common.^6^ Cardiovascular irAEs include myocarditis, pericarditis, and conduction abnormalities.^7^ Among all irAEs, myocarditis carries the highest fatality rate.^8^ ICI‐associated sarcoidosis (hereafter referred to as ICI‐sarcoidosis) is a rare irAE that manifests an average of 9 months after ICI initiation.^9^ One proposed mechanism is exaggerated Th1/Th17 T‐cell activation, leading to non‐caseating granuloma formation.^10^ ICI‐sarcoidosis resembles idiopathic sarcoidosis, including dyspnea, hypercalcemia, and hilar lymphadenopathy and/or pulmonary parenchymal disease.^10^ Cardiac involvement is rare.^11^

Sarcoidosis and myocarditis are two distinct forms of myocardial inflammation associated with ICI therapy. ICI‐cardiac sarcoidosis is defined by granulomatous inflammation of the myocardium, typically without cardiomyocyte necrosis, whereas ICI‐myocarditis is characterized by lymphocyte (CD4 and CD8 T‐cells) and macrophage infiltration with cardiomyocyte necrosis.^12^ Clinically, ICI‐cardiac sarcoidosis and ICI‐myocarditis manifest similarly with troponin elevation, atrioventricular block, tachyarrhythmias, heart failure, and, rarely, sudden cardiac death.^13^ ICI‐myocarditis frequently co‐occurs with ICI‐myositis and myasthenia gravis (known as the 3 M syndrome), while ICI‐cardiac sarcoidosis does not.^9^, ^14^, ^15^, ^16^

FDG‐PET/CT with resting myocardial perfusion protocol supports the diagnosis of ICI‐cardiac sarcoidosis but is not typically part of the ICI‐myocarditis evaluation. Evaluation of either form of ICI‐myocardial inflammation requires echocardiography and cardiac magnetic resonance imaging; coronary angiography is frequently done to exclude coincident coronary artery disease. Endomyocardial biopsy is instrumental for diagnosing both ICI‐cardiac sarcoidosis and ICI‐myocarditis, with unique pathological features that distinguish between these two conditions.^17^ The diagnostic sensitivity of endomyocardial biopsy for cardiac sarcoidosis is only 20%–30% because the granulomatous inflammation is most prominent in the left ventricular wall (a region not typically sampled during transvenous biopsy) and because early‐stage disease may lack well‐formed granulomas and instead feature histiocytic inflammation or scattered multinucleated giant cells.^18^ In this case, although well‐formed granulomas were not seen, the pattern of multinucleated giant cells, multi‐organ involvement, elevated 1,25‐dihydroxyvitamin D, and cardiac imaging findings collectively established the clinicopathologic diagnosis of cardiac sarcoidosis.

Current guidelines do not address the management of ICI‐cardiac sarcoidosis.^12^ Asymptomatic or mild ICI‐cardiac sarcoidosis usually does not require ICI discontinuation or immunosuppression.^10^ Symptomatic ICI‐cardiac sarcoidosis requires ICI interruption and prompt treatment with steroids to reduce the risk of arrhythmias but may not warrant permanent ICI discontinuation.^9^, ^16^, ^19^ Options for steroid‐refractory disease include methotrexate, infliximab, azathioprine, rituximab, and adalimumab.^19^

For ICI‐myocarditis, guidelines recommend permanent ICI discontinuation and pulse‐dose steroids (e.g., IV methylprednisolone 1 g/day). A non‐steroidal immunosuppressant (e.g., ruxolitinib, mycophenolate mofetil, or abatacept) is added within 24–48 h if there is no improvement in clinical status or biomarkers.^9^, ^15^, ^16^ In contrast, such rapid escalation is not required for ICI‐cardiac sarcoidosis.

Rapid clinical progression and hypercalcemia are not features of transthyretin cardiac amyloidosis, which led the clinicians to regard the amyloidosis as an incidental rather than explanatory finding. However, because amyloidosis represents a modifiable contributor to myocardial dysfunction, disease‐specific therapy was initiated alongside treatment for ICI‐cardiac sarcoidosis.

Novel therapies not only compel clinicians to learn new disease entities such as irAEs, but also challenge them to reconsider familiar conditions like sarcoidosis in new clinical contexts, revealing an old disease with a new twist.

## TEACHING POINTS

1. Immune checkpoint inhibitors (ICI) can trigger immune‐related adverse events (irAEs) that can affect multiple organs, including the skin, endocrine organs, gastrointestinal tract, lungs, muscles, liver, and heart.
2. ICI can produce distinct forms of myocardial inflammation—myocarditis and sarcoidosis—that present similarly but differ in pathophysiology, histology, and urgency of immunosuppressive therapy.
3. Endomyocardial biopsy has limited sensitivity for cardiac sarcoidosis. The absence of granulomas does not exclude the diagnosis when hypercalcemia, elevated 1,25‐dihydroxyvitamin D levels, and characteristic multiorgan involvement and imaging findings are present.

## CONFLICT OF INTEREST STATEMENT

The authors declare no conflicts of interest.

## Data Availability

The data that support the findings of this study are available on request from the corresponding author. The data are not publicly available due to privacy or ethical restrictions.
